# NMDARs mediate peripheral and central sensitization contributing to chronic orofacial pain

**DOI:** 10.3389/fncel.2022.999509

**Published:** 2022-09-27

**Authors:** Ya-Jing Liu, Yue-Ling Li, Zhong-Han Fang, Hong-Lin Liao, Yan-Yan Zhang, Jiu Lin, Fei Liu, Jie-Fei Shen

**Affiliations:** ^1^State Key Laboratory of Oral Diseases, National Clinical Research Center for Oral Diseases, National Center for Stomatology, West China School of Stomatology, Sichuan University, Chengdu, China; ^2^Department of Prosthodontics, West China Hospital of Stomatology, Sichuan University, Chengdu, China

**Keywords:** N-methyl-D-aspartate receptor, chronic orofacial pain, peripheral sensitization, central sensitization, ascending systems, descending system, sex differences

## Abstract

Peripheral and central sensitizations of the trigeminal nervous system are the main mechanisms to promote the development and maintenance of chronic orofacial pain characterized by allodynia, hyperalgesia, and ectopic pain after trigeminal nerve injury or inflammation. Although the pathomechanisms of chronic orofacial pain are complex and not well known, sufficient clinical and preclinical evidence supports the contribution of the N-methyl-D-aspartate receptors (NMDARs, a subclass of ionotropic glutamate receptors) to the trigeminal nociceptive signal processing pathway under various pathological conditions. NMDARs not only have been implicated as a potential mediator of pain-related neuroplasticity in the peripheral nervous system (PNS) but also mediate excitatory synaptic transmission and synaptic plasticity in the central nervous system (CNS). In this review, we focus on the pivotal roles and mechanisms of NMDARs in the trigeminal nervous system under orofacial neuropathic and inflammatory pain. In particular, we summarize the types, components, and distribution of NMDARs in the trigeminal nervous system. Besides, we discuss the regulatory roles of neuron-nonneuronal cell/neuron-neuron communication mediated by NMDARs in the peripheral mechanisms of chronic orofacial pain following neuropathic injury and inflammation. Furthermore, we review the functional roles and mechanisms of NMDARs in the ascending and descending circuits under orofacial neuropathic and inflammatory pain conditions, which contribute to the central sensitization. These findings are not only relevant to understanding the underlying mechanisms, but also shed new light on the targeted therapy of chronic orofacial pain.

## Introduction

Orofacial pain comprises multiple pain conditions of dentoalveolar, myofascial, temporomandibular joint (TMJ), musculoskeletal, neurovascular, and neuropathic origin affecting the oral, face, head, and neck areas (Anonymous, 2020). Patients with orofacial pain have been diagnosed with temporomandibular joint disorders (TMD), neuralgias, migraine, trauma, nerve injuries, inflammation, cancer, as well as various neurological/muscle disorders (Sessle, 2021). It markedly impacts the life quality of patients and brings a huge social and economic burden to medicine and society. Acute pain lasting less than 3 months is a protective mechanism that alerts the body to tissue damage and can be well controlled (Kuner and Kuner, 2021). while chronic pain present for more than 3 months is difficult to diagnose and treat because of its complex etiology and pathogenesis. Chronic orofacial pain, typically characterized by the spontaneous nature, allodynia, hyperalgesia as well as pain extra-territorial spread and referral, is often induced by nerve injury, orofacial tissue inflammation, and TMD (Dahlstrom and Carlsson, 2010; Shinoda et al., 2019; Sessle, 2021). Besides, it is also often accompanied by a variety of comorbidities, including depression, anxiety, stress, and sleep disorders (Sessle, 2021). Peripheral and central sensitization is considered to be involved in the above characteristics of chronic orofacial pain (Shinoda et al., 2019). Although lots of signal molecules, receptors, ion channels, and intracellular signal pathways involved in the activation or sensitization of the nociceptive afferents have been reported, the knowledge of the mechanisms underlying the initiation and maintenance of chronic orofacial pain is still limited, and more sufficiently and consistently effective treatments are needed to be found.

N-methyl-D-aspartate receptors (NMDARs, a subclass of ionotropic glutamate receptors) have been proved to be associated with abnormalities in the peripheral nervous system (PNS) and central nervous system (CNS), resulting in neuronal excitation and chronic pain manifestations (Petrenko et al., 2003; Hansen et al., 2018). Recently, increasing evidence has also investigated the role and mechanisms of NMDARs in the peripheral and central sensitization of chronic orofacial pain. The findings demonstrate that the up-regulation and activation of NMDARs in the trigeminal nervous system initiate intracellular cascades through calcium influx and activation of protein kinases, which in turn modulates cell membrane excitability and enhances nociceptive transmission, resulting in chronic orofacial inflammatory and neuropathic pain (Kimura et al., 2022; Zhang et al., 2022). Therefore, NMDAR has been proposed as a promising target for the treatment of chronic orofacial pain. Current studies have found that a variety of NMDAR antagonists and modulators have an analgesic effect on patients with pain, including ketamine, memantine, dextromethorphan, magnesium, etc. (Zhou et al., 2011; Shiiba et al., 2021). While there is still ambiguity about the efficacy of NMDAR antagonists on chronic pain because positive as well as negative effects of NMDAR antagonists on pain relief are found in clinical trials (Collins et al., 2010). In addition, because NMDARs exist widely in the nervous system and the physiological activity of NMDARs is essential to maintain the normal function of neurons, systemic administration of NMDAR antagonists may lead to a series of adverse events, including loss of consciousness, sedation, hallucinations, hearing and postural disorders, insomnia and so on (McCartney et al., 2004; Alviar et al., 2016). In order to provide a better understanding of the role and mechanisms of NMDARs in the regulation of chronic orofacial pain, and to facilitate further research on therapeutic strategies and new drugs targeting NMDARs, the types, components, and distribution of NMDARs in the trigeminal nervous system were discussed in this topical review. Besides, we discussed the regulatory roles and mechanisms of NMDARs in the peripheral and central sensitization in the ascending and descending circuits for chronic orofacial pain following nerve injury and inflammation.NMDARs are also involved in the regulation of acute pain which participates in the transition from acute to chronic pain and NMDAR antagonists can provide effective control of acute postoperative pain (Corder et al., 2013; Tognoli et al., 2020), while it is not the main point of this topical review.

PubMed, MEDLINE, Google Scholar, Scopus, and Embase were searched for the studies with English restriction focusing on the role of NMDARs in the peripheral and central nervous systems that contribute to chronic orofacial pain from the inception of the databases to Aug 9, 2022. The search strategy followed was: (NMDA receptor OR N-Methyl-D-aspartate receptor) AND (chronic pain OR neuropathic pain OR chronic inflammatory pain) AND (orofacial OR trigeminal) in Title/Abstract.

## Neural Circuits for The Signal Transmission of Orofacial Pain

**Figure 1:**
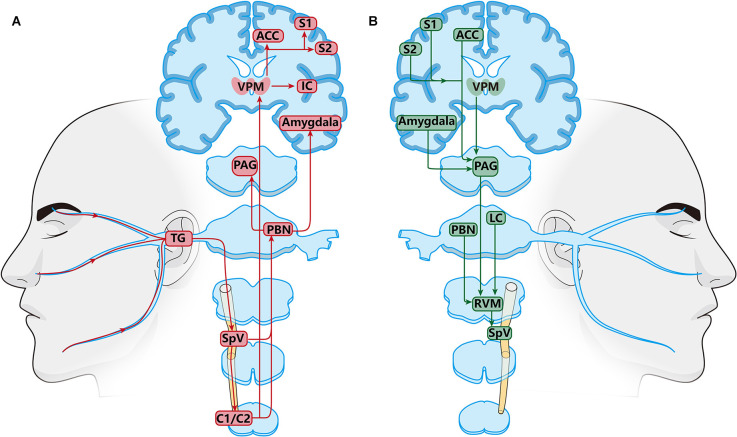
Schematic diagram of the orofacial pain modulation circuits. **(A)** Ascending nociceptive modulatory pathway. Neurons in the posteromedial thalamic nucleus (PBN) and thalamus (VPM) are key relays in ascending transmitting pathway. PBN and VPM receive nociceptive information from the SpVi/c, Vc, C1/C2 and project upward to the amygdala, anterior cingulate cortex (ACC), insular cortex (IC), the primary somatosensory cortex (S1), and secondary somatosensory cortex (S2). **(B)** Descending pain modulatory pathways. Periaqueductal gray (PAG), and ventrolateral medulla (VPM) are the main relays in the descending pain regulation pathway. The PAG receives regulatory information from the amygdala, VPM, ACC, S1, and S2, and finally transmits it down to the SpV through the RVM. TheRVM receives direct inputs from the PAG, thalamus, parabrachial region, and locus coeruleus (LC).

Peripheral orofacial noxious information is transmitted ultimately to the cortex and subcortical nuclei *via* three-order neurons (Figure 1A). The primary neurons are trigeminal ganglion neurons (TGNs), which are pseudounipolar neurons with peripheral and central processes. Their peripheral processes innervate peripherally to facial skin, oral mucosa, jaw, teeth, and deep tissues such as masseter to receive nociceptive, mechanical, and thermal sensations. Besides, their central processes project centrally to the spinal trigeminal nucleus (SpV) subdivided into oralis (Vo), interpolaris (Vi), and caudalis (Vc) located in the medullar oblongata and to the upper cervical spinal cord (C1/C2) to synapse with the secondary neurons (Olszewski, 1950; Fernandez-Montoya et al., 2017). Then, the secondary neurons in the SpV and C1/C2 decussate to the contralateral side and ascend to make synapses with the third-order neurons located in the higher nucleus in the thalamus and brain. Retrograde tract-tracing and anatomical studies demonstrate that the secondary neurons in Vi/c, Vc, and C1/C2 mainly project upward to the parabrachial nucleus (PBN) located in the pons and ventral posteromedial nucleus of the thalamus (VPM; Ren and Dubner, 2011; Li X. et al., 2017; Zhang et al., 2018). According to previous studies, it has been confirmed that the Vc-thalamic pathway and Vc-PBN pathway are two important ascending projection pathways that mediate orofacial pain (Nash et al., 2009; Saito et al., 2017). It is generally believed that the Vc-thalamic pathway is related to the sensory discrimination of pain, such as the intensity, nature, and location, while the Vc-PBN pathway is involved in pain-related emotions (Gauriau and Bernard, 2002; Bushnell et al., 2013; Chiang et al., 2019). Nociceptive information from the thalamus is further sent to the cortex (Castro et al., 2017). Neuroelectrical and neurochemical methods have confirmed that the brain regions related to pain perception included the anterior cingulate cortex (ACC), insular cortex (IC), the primary somatosensory cortex (S1), secondary somatosensory cortex (S2), and prefrontal cortex (PFC; Apkarian et al., 2005). Neurons in the PBN project upwards to the central amygdala (CeA), hypothalamus, the periaqueductal gray (PAG), and ventrolateral medulla (VPM), which are thought to be involved in processing the emotional components of pain (Missig et al., 2017; Liu et al., 2019; Wilson et al., 2019; Deng et al., 2020; Jiang et al., 2021). These findings on the anatomical and functional organization between the orofacial input and central termination explain chronic orofacial pain can be emotional and interact with autonomic and hormonal functions.

Nociceptive stimulation from orofacial areas not only activates the ascending nociceptive pathway but also triggers the descending pain regulation system (Figure 1B; Ren and Dubner, 2011). The descending regulation system is biphasic, including descending inhibition and descending facilitation system (Finnerup et al., 2021). The key integration centers of the descending circuit are the PAG and the rostral ventromedial medulla (RVM; Heinricher, 2016; Guo et al., 2022). The PAG receives descending nociceptive information from the somatosensory cortex, anterior cingulate cortex, hypothalamus, amygdala, and the dorsal medial prefrontal cortex, and then projects to the RVM (Floyd et al., 2000; Li and Sheets, 2018; Liang et al., 2020; Yin et al., 2020). In addition to PAG, the RVM plays a major role in the endogenous modulation of nociceptive transmission in the trigeminal nociceptive pathways in the ascending and descending nervous system. The RVM receives descending projections from several higher brain centers such as the PAG, PBN, locus coeruleus (LC), and hypothalamus (Behbehani and Fields, 1979; Willis, 1985; Fields et al., 1995; Ren and Dubner, 2011; Chichorro et al., 2017; Chen and Heinricher, 2019; Tang et al., 2021). The RVM can play a two-way role in pain regulation, including pain inhibition, and promotion. Earlier studies have found that electrical stimulation of RVM promotes nociceptive response at a lower intensity, while higher intensity stimulation has an antinociceptive effect (Zhuo and Gebhart, 1992; Sivanesan et al., 2019).

The joint regulation of ascending and descending pathways together forms the nociceptive sensation. Under physiological conditions, the ascending pathway is in balance with the endogenous descending pathway (Mills et al., 2020). In addition, nerve injury and inflammation trigger changes in molecules, receptors, ion channels, and neuroplasticity in the neural circuits of the trigeminal nervous system, leading to the imbalance between ascending and descending pain regulation system, which may be the basis of pathological pain (Ren and Dubner, 2011). Among the various potential targets, NMDARs have been reported to play a crucial role in the peripheral and central sensitization contributing to chronic orofacial pain (Wang et al., 2006; Ren and Dubner, 2011; Zhang et al., 2022).

## Characteristics of NMDARs

NMDAR, a type of ionotropic glutamate receptor, consists of four subunits [two NR1 subunits and two NR2 (NR2A-D) or NR3 (NR3A-B) subunits], which together form a central ion channel pore (Sanz-Clemente et al., 2013a; Hansen et al., 2018). Seven genes encode the NMDAR subunits: a single *GRIN1* gene encodes the NR1 subunit including eight NR1 distinct isoforms four *GRIN2* genes encode NR2A-D, and two *GRIN3* genes encode NR3A-B (Hardingham and Bading, 2010; Sanz-Clemente et al., 2013a). The two NR1 subunits, the binding site of glycine orD-serine, are obligatory in all functional NMDARs (Fernandez-Montoya et al., 2017; Hansen et al., 2018; Wu and Tymianski, 2018). The functional consequences of the gating reaction mechanism of NMDARs are strongly dependent on the identity of the four types of NR2 subunits (NR2A-D), the binding site of glutamate (Monyer et al., 1992; Wu and Tymianski, 2018). However, subunit assembly and physiological roles of the glycine-binding NR3 subunits remain elusive (Cavara and Hollmann, 2008; Henson et al., 2010). The detailed structure of NMDAR subunits has been described in a previous review (Hansen et al., 2018). The NR1 subunits combined with either NR2A or NR2B are the most widely expressed NMDARs in the nervous system (Wu and Tymianski, 2018).

Notably, NMDARs have several unique properties, including a voltage-dependent block by Mg^2+^, high permeability to Ca^2+^, and the requirement for binding of two coagonists (glutamate and glycine/D-serine) for channel activation, which can distinguish them from other glutamate ionotropic receptors (Traynelis et al., 2010). It is well-known that excitatory synaptic transmission is mainly regulated by the pre- and post-synaptic NMDARs. NMDARs are permeable to Na^+^, K^+^, and Ca^2+^ to regulate the membrane potential (Sanz-Clemente et al., 2013a; Hansen et al., 2018). NMDAR pore is strongly blocked by extracellular Mg^2+^ at the resting potential state, which can be released by neural depolarization or corresponding NMDAR agonists, resulting in a massive influx of Ca^2+^ (Traynelis et al., 2010). Then, the Ca^2+^ influx through NMDARs serves as a trigger for intracellular cascade signal activation such as the activation of Ca/calmodulin-dependent protein kinases II (CaMKII; Lee et al., 2012), which dramatically modifies neuronal functional properties and synaptic plasticity including short-term or long-term changes in synaptic strength (Hansen et al., 2018). Under physiological conditions, presynaptic NMDARs are not functionally active in regulating neurotransmitter release. However, in opioid-induced hyperalgesia and chronic neuropathic pain conditions, presynaptic NMDARs become tonically active and are stimulated by endogenous glutamate (Deng M. et al., 2019).

NMDAR subtypes confer different gating and permeation properties to the channel. One important property of NR1 is reduced agonist potency (Traynelis et al., 1998). In addition, NR1 can alleviate the inhibition of NMDAR function by NR2B-selective antagonist (Durand et al., 1992; Yi et al., 2018). These findings demonstrate the interactions between NR1 and NR2 subunits. NR2A and NR2B have unique characteristics, NR2A-containing receptors have faster activation/inactivation kinetics and a higher channel opening probability than the receptors containing NR2B subunits (Erreger et al., 2005; Sanz-Clemente et al., 2013a). NR2C-containing receptors show relatively unique channel properties, including low conductance, open probability, and sensitivity to Mg^2+^ (Farrant et al., 1994). NR2D is characterized by its expression early in development and its extremely slow decay time (Deng M. C. et al., 2019). Unlike NR2 subunits, NR3 binds to glycine and not to glutamate (Henson et al., 2010). The NR3 subunits function mainly as modulatory subunits that can reduce the vulnerability to Mg^2+^ block and attenuate the Ca^2+^ permeability of NMDARs (Cavara et al., 2010). NMDARs containing exclusively NR1/NR3 subunits can act as excitatory glycine receptors, which are impermeable to calcium.

Interestingly, recent studies have confirmed that NMDAR is mechanically sensitive (Casado and Ascher, 1998; Belin et al., 2022). In the model of stretch-induced neuronal injury, the Mg^2+^ blockage of the NMDAR channel significantly decreased and the NMDAR current increased (Zhang et al., 1996). Subsequent studies found that artificial lipid bilayer stretching can lead to the same phenomenon, indicating that mechanical energy is sufficient to regulate the activity of the NMDAR channel and is related to the decrease of Mg^2+^ blockage (Kloda et al., 2007). In addition to mechanical sensitivity, the latest study reported that NMDAR-mediated calcium influx in the absence of glutamate by applying shear force in cultured astrocytes *in vitro*, which indicated that mechanical stimulation itself can activate NMDAR currents (Maneshi et al., 2017). The oral and maxillofacial region receives abundant mechanical stimulation, and the NMDAR located in the peripheral nerve fibers may be more or less exposed to changing mechanical forces, therefore, understanding the regulation of mechanical sensitivity on NMDARs may be helpful to provide improved methods for chronic orofacial pain treatment.

## The Changes in The Expression and Activation of NMDARs in The Neural Circuits Are Associated with Orofacial Pain Conditions

NMDARs are widely expressed in the PNS and CNS, such as the TG, dorsal root ganglion (DRG), spinal cord, brainstem, hippocampus, cerebellum, cortex, etc. (Dedek and Hildebrand, 2022). Research on the expression of NMDARs in the TG has found that about one-third of TG neurons express functional NMDARs, and the main subunits include GluN1, GluN2A, and GluN2B subunits (Fernandez-Montoya et al., 2016). And 99% of ganglion cells express GluN1, about 30% of TG neurons contain GluN2B subunits, and GluN2A subunits are expressed between 16% and 80% in different studies (Guerrero-Toro et al., 2022). The changes in the expression of NMDARs in the neural circuits of PNS and CNS are related to chronic orofacial pain including orofacial neuropathic pain, craniofacial musculoskeletal pain, and inflammatory pain induced by nerve injury and inflammation of the facial area, TMJ, and masseter (Li Y.-L. et al., 2021; Kimura et al., 2022; Zhang et al., 2022). Evidence supports that NMDARs exist in the peripheral terminals of small and large diameter primary afferent fibers in orofacial tissues such as masseter muscle, face, and TMJ; activation of NMDARs on the peripheral nerve endings can contribute to inflammatory pain (Yu et al., 1996; Cairns et al., 2003; Ro, 2003; Park et al., 2011). Although the expression of NMDARs in the periphery is elevated during inflammation both in animals and humans, studies on the relationship between chronic orofacial neuropathic pain and changes in the expression of NMDARs in the peripheral terminals are still lacking. Recently, several previous studies have focused on the role of NMDARs in the TG in the development of chronic orofacial neuropathic and inflammatory pain. For example, the expression of NR1 in the TG (expressed in the TGNs and SGCs) is upregulated under the condition of orofacial ectopic pain induced by inferior alveolar nerve transection (IANX; Fu et al., 2021; Li Y.-L. et al., 2021). Besides, p-NR2B is significantly upregulated in the TG of mice under the inflammatory orofacial pain condition induced by complete Freund’s adjuvant (CFA) injection into the whisker pad and orofacial neuropathic pain condition induced by INAX (Zhang et al., 2022). Moreover, conditional knockout NR2B in the TG alleviates the mechanical allodynia induced by inflammation and IANX (Zhang et al., 2022).

In the CNS, the expression of NR1, IL-6, and NF-κB are elevated following TMJ inflammation (Wang et al., 2009). Intracisternal injection of an IL-6 antiserum or NF-κB inhibitor (PDTC) can prevent both the upregulation of NR1 in the ipsilateral SpVc and pain behavior (Wang et al., 2009). Moreover, intracisternal IL-6 administration in naïve rats induces the NR1 upregulation and pain behavior similar to that after TMJ inflammation (Wang et al., 2009). These results indicate that the upregulation of IL-6 and NF-κB after inflammation of the unilateral TMJ region is a critical regulatory mechanism for the expression of NR1 in the ipsilateral SpVc, which contributed to the development of TMJ pain behavior in rats. p-NR2B is also upregulated in the SpVc after IANX and CFA-induced facial inflammation (Zhang et al., 2022). Moreover, TMJ arthritis can increase the expression of mRNA for all NMDAR subunits and p-NR1 in the SpVc, which is involved in the activation of astrocytes, resulting in chronic orofacial pain (Wang et al., 2012a; Cavalcante et al., 2013).

NMDARs are also expressed in astrocytes. Studies of the last two decades have provided evidence for the expression of all major NMDAR subunitsin astrocytes and the subunit composition of the receptor showing variation with tissue preparation and physiological context (Schipke et al., 2001; Dzamba et al., 2013; Jimenez-Blasco et al., 2015). The biophysical and pharmacological properties of the astrocytic NMDARs strongly suggest that they have AN atri-heteromeric structure composed of GluN1, GluN2C/D, and GluN3 subunits (Palygin et al., 2011; Hogan-Cann and Anderson, 2016). Accordingly, these NMDARs have distinct functional characteristics, including weak susceptibility to channel Mg^2+^ blockade, even at negative restingmembrane potentials, and reduced Ca^2+^ permeability (Hogan-Cann and Anderson, 2016). Astrocytic NMDARs may also be involved in neuroinflammatory processes (Hogan-Cann and Anderson, 2016). Astrocytic NMDARs may contribute to morphological transformations characteristic of reactive astrogliosis (Ting et al., 2009), and may mediatet he release of proinflammatory cytokines (Gerard and Hansson, 2012). Compared to neuronal NMDARs, astroglial NMDARs have different pharmacological properties with higher affinity to UBP141, memantine, and D-AP5, and lower sensitivity to Mg^2+^ and ifenprodil which may be due to their incorporation of GluN2C or GluN2D subunits (Palygin et al., 2011). The other important finding of this study is the difference in the affinity of glial and neuronal NMDARs to memantine at physiological concentrations of Mg^2+^ (Palygin et al., 2011). These findings above may be veryuseful for elucidating the mechanisms of neuron-glia communications and the development of novel therapeutic agents specifically targeting glial signaling.

However, there is still little known about the changes in the expression of NMDARs in the higher central nucleus such as PAG and RVM under chronic orofacial pain. According to the studies about chronic pain occurring in the other region such as the arms, legs, and lumbar region, NR2B in ACC is upregulated (Wu et al., 2005). Notably, there is a study that has reported that NR2A and NR2D mRNAs in the contralateral thalamus show an increase in dental pulp inflammation and it can be reduced by MK-801 (an NMDAR antagonist) application (Kaneko et al., 2011). It is reasonable to speculate that the NMDARs in the ascending and descending nervous system of the CNS also play a vital role in chronic orofacial pain. In conclusion, the above findings demonstrate that NMDARs are an important mediator involved in the peripheral and central mechanisms of chronic orofacial pain.

## The Regulation in The Expression and Activation of NMDARs

**Figure 2:**
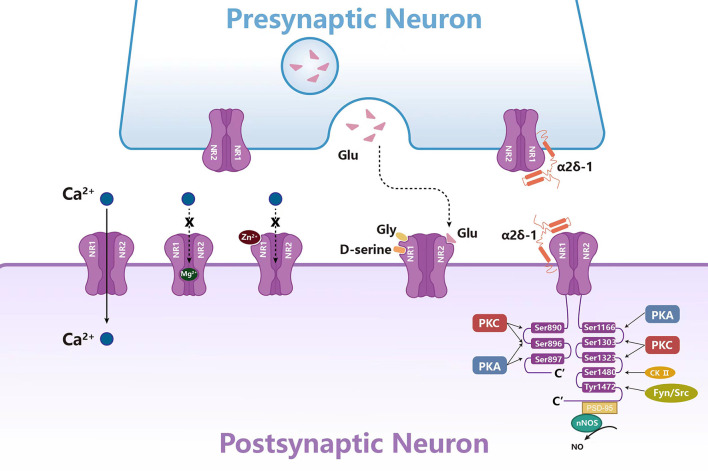
Molecules in NMDAR regulation in the synaptic transmission. The activity of NMDARs is regulated by a variety of ions, amino acid neurotransmitters, protein kinases, and scaffold proteins. Mg^2+^ and Zn^2+^ play an antagonistic role by blocking the channel of NMDARs. Gly, Glu, and D-serine can activate NMDAR together. Protein kinases such as PKC, PKA, CK-II, Fyn, and Src can phosphorylate NR1 or NR2. Scaffold proteins α2δ-1 and PSD-95 regulate NMDAR activity by directly binding to NMDARs.

The functional status of NMDARs is closely related to pain. Current studies have found that NMDARs are directly or indirectly regulated by a variety of ions, amino acid neurotransmitters, protein kinases, and scaffold proteins (Figure 2; Bereiter et al., 2000; Li X. et al., 2017; Zhang et al., 2018; Deng et al., 2020). These intermolecular interactions regulate the activity, membrane transport, localization, and signal transduction of NMDARs, thereby affecting neuronal excitability and pain.

### Regulation of NMDAR function by extracellular ions

The functional state of NMDARs in the central nervous system is mainly regulated by Mg^2+^. As a physiological antagonist of NMDARs, Mg^2+^ can block the calcium influx of the channel and maintain the resting membrane potential (Kulik et al., 2021). The depolarization process removes the Mg^2+^-induced blockade of NMDARs in a voltage-dependent manner, resulting in hyperexcitability and consequently a sustained state of depolarization and increased synaptic strength (Johnson and Ascher, 1990). And oral administration of MgCl_2_ dissolved in mineral water inhibits expression of NR2B mRNA and p-NR1 and increases expression of NR3 mRNA in the SpVc, while Mg^2+^ deficiency increases expression of both NR1 and NR3 mRNAs and p-NR1 in the SpVc, contributing to the orofacial pain with TMJ arthritis (Cavalcante et al., 2013). Similarly, Mg^2+^ deficient rats develop mechanical hyperalgesia, which is reversed by NMDAR antagonists (Begon et al., 2001). Preclinical studies have found that the combination of Mg^2+^ and opioids can relieve neuropathic pain in diabetic rats by reducing the level of PKA in the PAG and increasing opioid receptor activity, indicating that Mg^2+^ can be used as a supplement for the treatment of pain (Rondon et al., 2010; Kulik et al., 2021). In addition to Mg^2+^, *in vitro* studies on mouse hippocampal neurons have confirmed that Zn^2+^ can selectively block NMDAR-mediated central neuron excitation (Westbrook and Mayer, 1987; Choi and Lipton, 1999; Morabito et al., 2022). Zn^2+^ can inhibit NMDAR by high affinity, voltage-independent, and low affinity, voltage-dependent (Fayyazuddin et al., 2000; Rachline et al., 2005). Nozaki et al. have found that the blocking effect of Zn^2+^ disappeared in the mice with NR2A knockout in hippocampus and spinal cord, resulting in thermal hyperalgesia (Nozaki et al., 2011). In addition, the analgesic effect of exogenous Zn^2+^ on inflammatory and pathological pain disappeared in NR2A knockout mice. Therefore, the interaction of Zn^2+^-NR2A may be one of the molecular bases for the occurrence and development of pain (SafiehGarabedian et al., 1996; Liu et al., 1999; Abdelrahman and Hackshaw, 2021).

### Amino acids and NMDAR activation

NMDARs require co-activation of glutamate and glycine/D-serine: glutamate binds to the NR2 subunit and glycine/D-serine binds to the NR1 subunit to activate this channel (Chou et al., 2020). Glutamate and glycine can trigger conformational changes in the NMDAR ligand-binding domain, resulting in the opening of NMDAR channel pores, allowing calcium influx and depolarizing membrane potential, and mediating neuronal excitation (Yu and Lau, 2018). Besides, serine is also considered to be the endogenous ligand of the glycine site of the NMDAR. It is found that activated astrocytes may release endogenous D-serine to activate NMDAR, resulting in pain hypersensitivity (Miraucourt et al., 2011). Exogenous D-serine increases the phosphorylation of spinal cord PKC-dependent NR1 in Ser896 and promotes NMDAR-mediated mechanical hyperalgesia after nerve injury, which was alleviated by pretreatment with PKC inhibitors (Choi et al., 2017). In addition to directly regulating pain, further studies have confirmed that endogenous D-serine in rat rostral anterior cingulate cortex can also regulate pain-related negative emotion mediated by NMDAR (Ren et al., 2006). Therefore, reducing D-serine release or selectively inhibiting the glycine site of NMDAR may prevent emotional disturbance caused by chronic pain.

### Phosphorylation of NMDARs

It has been reported that NMDAR phosphorylation is observed in both neuropathic and inflammatory pain (Pan et al., 2022; Zhang et al., 2022). NMDARs can be phosphorylated at different sites by a variety of kinases. It has been confirmed that the NR1 subunits can be phosphorylated by PKC at Ser890 and Ser896, and by PKA at Ser896 or Ser897 site (Tingley et al., 1993). In addition to the NR1 subunits, NR2 subunits can also be phosphorylated. The C-terminals of NR2A and NR2B are considered to be the main target of protein-protein interaction and phosphorylation, which contains multiple amino acid sites and can be phosphorylated by PKA, PKC, casein kinase II (CK2), Src, and Fyn tyrosine kinases (Tang et al., 2001; Cull-Candy and Leszkiewicz, 2004; Groc et al., 2009; Zhang et al., 2022). PKA can phosphorylate NR2B at Ser1166, which affects Ca^2+^ permeability in the spinal cord (Murphy et al., 2014). Notably, it is reported that subcutaneous administration of IL-1β in the vibrissa pad of rats induces orofacial mechanical allodynia by sensitizing peripheral NMDARs through PKA-mediated signaling in the large diameter primary afferent nerve fibers (Kim M. J. et al., 2014). On the other hand, activation of PKC can phosphorylate Ser1303 and Ser1323 at the C-terminal of NR2B (Liao et al., 2001), which potentiate the NMDAR currents by increasing the probability of channel openings and by reducing the voltage-dependent Mg^2+^ block of NMDAR channels in the isolated TGNs (Chen and Huang, 1992). CK2 is a serine/threonine protein kinase, which can mediate the phosphorylation of NR2B at Ser1480, drive NR2B endocytosis, reduce synaptic NR2B, and increase synaptic NR2A (Ye et al., 2012; Sanz-Clemente et al., 2013b; Chen et al., 2014). CK2 inhibitors can block the increase of NMDAR current in spinal dorsal horn neurons induced by nerve injury in rats, and inhibition of CK2 or CK2 gene knockout at the spinal cord level can significantly reverse the hyperalgesia caused by nerve injury (Chen et al., 2014). Src and Fyn can promote the phosphorylation of NR2B in the spinal dorsal horn at Tyr1472, resulting in up-regulation of NMDAR activity and membrane localization (Yang et al., 2011; Lai et al., 2016). Subsequent studies found that inhibition of Src activity can reduce Src-mediated NR2B phosphorylation, reduce postsynaptic density NR2B accumulation, and ultimately improve inflammatory pain (Guo et al., 2002; Suo et al., 2013). Similarly, after neuropathic injury and intrathecal activation of Fyn, the phosphorylation of NR2B in the spinal dorsal horn increased at Tyr1472 and led to hyperalgesia, which could be blocked by pre-inhibition of Fyn (Hildebrand et al., 2016; Nie et al., 2021). The membrane translocation of NMDARs is regulated by PKA and PKC phosphorylation. When PKA and PKC phosphorylate Ser897 and Ser896, respectively, the expression of NMDARs in the membrane increases (Scott et al., 2003). In summary, in the CNS, NMDAR phosphorylation strengthens the interaction between NMDAR and the postsynaptic density, which plays an essential role in the induction of long-term potentiation (LTP), a representative form of synaptic plasticity under chronic neuropathic pain conditions (Kronschlager et al., 2016).

### Protein interaction and NMDAR activation

Recent studies have found that some intracellular proteins may be involved in NMDAR transport, synaptic localization, and activity regulation through interaction with NMDARs. Postsynaptic density protein-95 (PSD-95) is a scaffold protein that can cause a series of downstream molecular events that can cause pain by interacting with NMDARs (Chen et al., 2021a,b). It is generally believed that PSD-95 couples nNOS to NMDARs through the PDZ domain to form an NMDAR-PSD95-nNOS complex, which produces NO and leads to central sensitization (Lee et al., 2015; Li J. et al., 2021). NOS1 adaptor protein (NOS1AP) can bind to the N-terminal region of nNOS containing PDZ (Jaffrey et al., 1998). Current studies have found that NOS1AP mediates the inhibition or activation of NMDAR-driven nNOS (Li et al., 2013; Weber et al., 2014). The activation of NMDARs increases the connection between nNOS and NOS1AP, while the destruction of nNOS-NOS1AP interaction can inhibit the behavioral hypersensitivity in the model of neuropathic pain induced by chemotherapeutic drugs and trauma (Lee et al., 2018). Several experiments have confirmed that TAT-NR2B9c, IC87201, and ZL006 can block the central sensitization triggered by NMDARs by disrupting the interaction between NMDARs and PSD95, which can produce antinociceptive effects in both inflammatory and neuropathic pain models (Pedersen and Gjerstad, 2008; Chen et al., 2014; Peltoniemi et al., 2016; Li S. et al., 2017). α2δ-1, a subunit of voltage-activated calcium channel, is another powerful regulator of NMDARs. It promotes neuropathic pain by forming a complex with NMDARs and promoting the synaptic transport of NMDARs (Chen et al., 2018). And gabapentin can relieve pain hypersensitivity by inhibiting the synaptic transmission of the α2δ-1-NMDAR complex (Huang et al., 2020; Fu et al., 2021). It has been proved that gabapentin can be used to treat neuropathic pain and epilepsy (Moore et al., 2014). Similarly, NR1 can also physically and functionally interact with α2δ-1 in the TGNs and activated SGCs after trigeminal nerve injury, inhibition of the formation of α2δ-1-NR1 complex can alleviate mechanical allodynia in the whisker pad of rats treated with IANX (Fu et al., 2021). Furthermore, NR1 and NR2B form protein-protein complexes with TRPV1 in the TG, contributing to the mechanical hyperalgesia induced by the injection of NMDA into the masseter muscle *via* CaMKII and PKC signaling cascades (Lee et al., 2012). NMDAR-protein interaction is essential for central sensitization and the development and maintenance of pain, thus, interference with the protein-protein interaction downstream of NMDARs provides a potential target for the development of anti-allodynic drugs. Several experiments have confirmed that interfering peptides such as TAT-NR2B9c and TAT-GESV can produce anti-pain effects by disrupting the interaction between PSD95-nNOS and nNOS-NOS1AP, respectively, without causing dyskinesia or changing the normal nociceptive sensation (Pedersen and Gjerstad, 2008; Chen et al., 2014; Peltoniemi et al., 2016; Li S. et al., 2017). Some small molecular compounds such as IC87201 and ZL006 can destroy the interaction between NMDARs and PSD95 but have no significant effect on the interaction between PSD95 and other proteins (Tochio et al., 2000; Florio et al., 2009). Similarly, gabapentin and α2δ-1 TAT peptide can relieve pain allergy by blocking the binding of α2δ-1 to NMDARs (Moore et al., 2014; Huang et al., 2020; Fu et al., 2021). Blocking the protein-protein interaction downstream of NMDARs is a valuable therapeutic strategy, which can inhibit neuropathic pain without affecting the normal function of NMDARs (Zhou et al., 2010).

## The Functional Role and Mechanisms of NMDARs in Regulating Peripheral Sensitization of Chronic Orofacial Pain

Evidence indicates subcutaneous injection of glutamate mechanically sensitizes rat facial cutaneous mechanoreceptors through activation of peripheral NMDARs (Gazerani et al., 2010). The subcutaneous injection of PPPA, a competitive NR2A antagonist, and Ro 25-6981, a selective NR2B antagonist, can inhibit the nociceptive scratching behavior produced by the injection of formalin (Park et al., 2011). These results suggest NR2 subunits participate in the transmission of nociceptive information in peripheral tissues, indicating peripheral NMDAR antagonists may be considered for chronic orofacial pain. Besides, it is reported that stimulation of peripheral NMDARs also causes the release of the neuropeptides, such as substance P (SP) and calcitonin gene-related peptide (CGRP), from peripheral nerve terminals (McRoberts et al., 2001). These neuropeptides further contribute to the initiation and development of chronic orofacial pain induced by nerve injury and inflammation. In addition, masseter injection of MK801, an NMDA receptor antagonist, attenuates nocifensive behaviors in lightly anesthetized rats and decreases c-fos expression in the SpVc induced by the masseteric injection of mustard oil (Ro, 2003; Ro et al., 2004), which demonstrates that the activation of NMDARs on the peripheral terminals is involved in the neuroplastic changes in the synaptic transmission in the SpVc under orofacial inflammatory conditions. Taken together, these results support the suggestion that the orofacial allodynia or hyperalgesia due to increased peripheral terminal NMDARs mediates the release of neuropeptides under the condition of nerve injury and inflammation.

**Figure 3:**
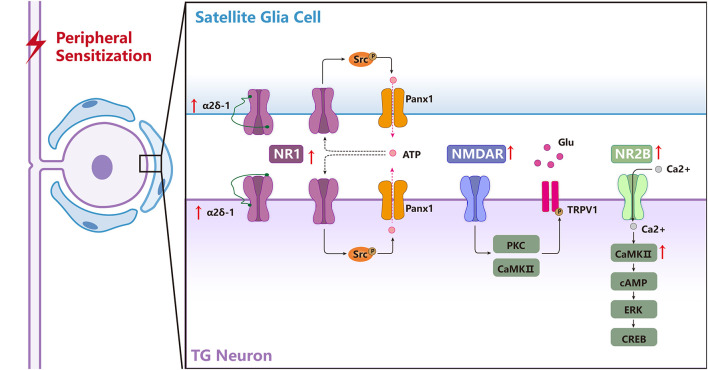
The biological mechanisms of NMDARs in the TG underlying regulating peripheral sensitization of orofacial pain. NMDARs are involved in the activation of chronic orofacial pain-related intracellular signaling pathways and the communication among the neuronal and satellite glial cells (SGCs) in the TG. NR1 forms a complex with α2δ-1 in the TGNeurons (TGNs) and activates SGCs, and the α2δ-1-NR1 complex is upregulated after trigeminal nerve injury, α2δ-1 may regulate NR1 expression through PKC. NR1-Src-Panx1 signal pathway distributes in the TGNs and SGCs contributes to orofacial neuropathic pain following trigeminal nerve injury. Activation of NMDARs results in phosphorylation of TRPV1, primarily at serine residues through the activation of PKC and CaMKII pathways in rat trigeminal sensory neurons, and NMDAR and TRPV1 interactions are essential for the development of mechanical hyperalgesia in the masseter muscle after inflammation. NR2B/CaMKII acts as an upstream cascade to facilitate cAMP production and ERK-CREB activation in the TG after inflammatory and nerve injury.

Peripheral sensitization in the TG is a key mechanism underlying chronic orofacial pain induced by trigeminal nerve injury or orofacial inflammation. NMDARs have been reported to be involved in the peripheral sensitization in the TG by triggering the activation of intracellular signaling pathways and the communication between neuronal and SGCs (Figure 3; Fu et al., 2021; Li Y.-L. et al., 2021; Zhang et al., 2022). Following a chronic constriction injury of the inferior orbital nerve (CCI-ION), glutamate expression increases in the TG (Kung et al., 2013), further activating the NMDARs. In addition, glutamate increase occurs in the somata of neurons with injured axons as well as in the somata of neighboring uninjured neurons (Kung et al., 2013), indicating a cross-talk among TGNs. Besides, the p-NR2B expression in the TG is elevated following IANX or orofacial inflammation, indicating NR2B-containing NMDARs are activated under chronic orofacial pain conditions (Zhang et al., 2022). Activated NR2B can trigger the activation of CaMKII through the Ca^2+^ influx *via* autophosphorylation of the inhibitory domain in Thr286 to facilitate the activation of the cAMP-ERK-CREB signal pathway, resulting in the upregulation of transcriptional regulators and/or pain-related proteins, thus contributing to the development and maintenance of orofacial allodynia (Zhang et al., 2022). Besides, NR1 subunits also contribute to orofacial ectopic pain induced by IANX through activation of Src (a nonreceptor-type protein tyrosine kinase), further resulting in the upregulated expression of pannexin1 (Panx1), a subtype of pannexin family that is permeable to molecules smaller than 1 kDa, such as ions and neurotransmitters, for example, the algogenic ATP molecules (Fu et al., 2021; Li Y.-L. et al., 2021). Moreover, inhibition of Panx1 can in turn suppress the protein expression of NR1 due to ATP release through the Panx1 channel, which demonstrates a positive feedback loop on the expression of NR1 after trigeminal nerve injury (Li Y.-L. et al., 2021). Under masseter inflammation conditions, activation of NMDARs in the TG results in the phosphorylation of TRPV1, primarily at serine residues through the activation of PKC and CaMKII but not PKA pathways in rat TGNs (Lee et al., 2012). Meanwhile, NMDARs and TRPV1 interactions are essential for the development of mechanical hyperalgesia in the masseter muscle (Lee et al., 2012).

## The Functional Role and Mechanisms of NMDARs in Central Sensitization

**Figure 4:**
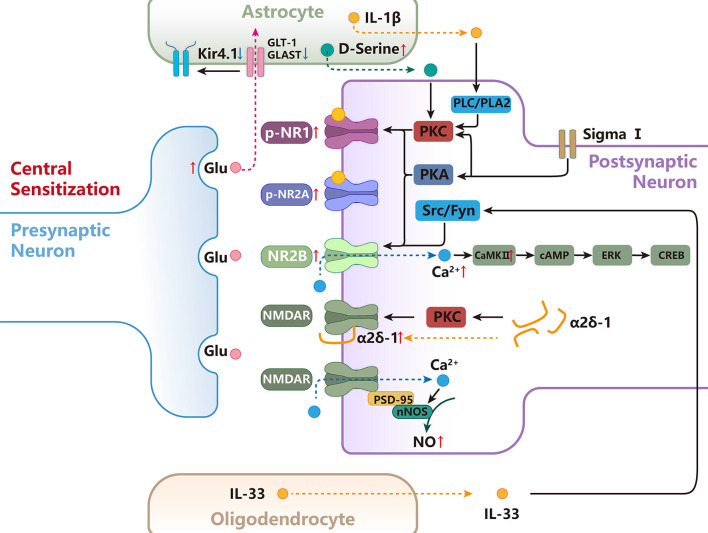
The functional role and mechanisms of NMDARs in regulating central sensitization of orofacial pain. D-Serine released from astrocytes increases PKC-dependent NR1 phosphorylation, contributing to central sensitization. Activation of the sigma-1 receptor enhances NMDA-induced pain *via* PKC- and PKA-dependent phosphorylation of the NR1 subunit. Src and Fyn can promote the phosphorylation of GluN2B at Tyr1472, resulting in up-regulation of NMDAR activity and membrane localization.NR2B/CaMKII acts as an upstream cascade to facilitate cAMP production and ERK-CREB activation after inflammatory and nerve injury in the SpVc. NMDAR forms a heteromeric complex with α2δ-1 in the spinal cord, and the α2δ-1-NMDAR interaction promotes surface trafficking and synaptic targeting of NMDAR. α2δ-1 can also indirectly regulate NMDAR through PKC.PSD-95 couples nNOS to NMDAR to form an NMDAR-PSD95-nNOS complex, which produces NO and leads to central sensitization. Astrocytes are responsible for the majority of glutamate uptake, which occurs mainly through two transporters, GLT-1 and GLAST, and Kir4.1 functions in the facilitation of astrocytic glutamate uptake. IL-33 act on the postsynaptic neuron to promote the phosphorylation of NR2B through activation of Fyn. the intracellular signal pathway of PLC, PLA2, and subsequent PKC activation is involved in the astrocyte-derived IL-1β-induced NR1 phosphorylation.

Nerve signals arising from sites of tissue or nerve injury lead to long-term increases in excitability and plasticity in the central nervous system often referred to as central sensitization (Ren and Dubner, 2011). NMDARs are well-known to be required for triggering central sensitization under chronic pain conditions by removal of the voltage-dependent magnesium block and phosphorylation (Ren and Dubner, 2011). In addition, it is well-known that the excitatory synaptic transmission is mainly regulated by the pre and postsynaptic NMDARs after being activated by glutamate. In the CNS, activation of NMDARs initiates intracellular cascades through calcium influx and activation of protein kinases, which in turn modulates cell membrane excitability, synaptic hyperexcitability, long-term plasticity, and enhances nociceptive transmission (Figure 4; Woolf and Mannion, 1999).

It is reported that presynaptic NMDAR activation can regulate the release of downstream neurotransmitters, thereby affecting hyperalgesia (Bardoni et al., 2004). Presynaptic NMDARs are known to facilitate the release of glutamate, SP, and BDNF from C-type primary nociceptive fibers (Larsson and Broman, 2011; Bardoni, 2013). Liu et al. (1997) found that NMDAR activation can lead to persistent pain by promoting the release of SP and glutamate. The changes in ions and neurotransmitters induced by NMDAR activation can change the excitability and synaptic plasticity of neurons (Ikeda et al., 2003). Repeated stimulation of peripheral C fibers can induce a gradually increased response of WDR neurons, resulting in overexcitation, a phenomenon known as windup (Mendell and Wall, 1965; Woda et al., 2004). Studies have found that windup actually depends on the activation of NMDARs (Woda et al., 2004; Dedek and Hildebrand, 2022). Under pathological conditions, the dysfunction of NMDAR-dependent windup inhibition may contribute to the central sensitization of the pain system (Mendell, 2022). Because of some characteristics that windup can induce central sensitization, it has been used as an experimental model to explore the mechanism of central sensitization (Coste et al., 2008). Long-term potentiation (LTP) was another important form of synaptic plasticity and it was considered a potential mechanism of hyperalgesia (Li X.-H. et al., 2021). Activation of NMDARs can further mediate LTP in neurons and induce hyperalgesia. It is reported that NMDAR-dependent LTP is widely found in synapses of the spinal dorsal horn and cortex (Zhuo, 2014; Li et al., 2019). Animal studies have confirmed that the induction of *LTP in vivo* requires the activation of NMDAR (Liu and Sandkühler, 1995; Kim et al., 2015). Besides, inhibition of NMDAR-dependent LTP has an analgesic effect (Benrath et al., 2005; Li and Baccei, 2016).

### The central sensitization involved NMDARs in the spinal trigeminal nucleus

NMDARs have been found to play a role in regulating pain in several key relays in the ascending pain regulation pathway. Currently, the role and mechanisms of NMDARs related to chronic orofacial pain are mainly focused on the Vi/Vc transition zone (SpVi/c) and SpVc. The intracisternal administration of NR2A or NR2B subunit antagonists inhibits the nociceptive behavior produced by the injection of formalin into the vibrissa pads of rats (Yang et al., 2010). Similar to the role of NMDARs in the peripheral sensitization in the TG, it is also reported that NR2B/CaMKII act as an upstream cascade to facilitate cAMP production and ERK-CREB activation in the SpVc after inflammatory and nerve injury which increase neuronal excitability, contributing to the development and maintenance of central sensitization (Zhang et al., 2022). It has been reported that oligodendrocyte-derived IL-33 can act on the receptor suppression of tumorigenicity 2 on the postsynaptic neuron to promote the phosphorylation of synaptosomal NR2B through activation of Fyn and potentiate NR2B-containing NMDAR-mediated synaptic currents in the SpVc, which facilitates orofacial neuropathic pain induced by ION injury (Kimura et al., 2022). These findings demonstrate that NMDARs are involved in the interaction between oligodendrocyte-neuron interactions, resulting in the development of orofacial neuropathic pain. In addition, D-serine, an endogenous and mandatory co-agonist for the NMDAR glycine site in mammalian brains, is predominantly expressed in astrocytes and increases in the SpVc after CCI-ION (Wolosker, 2006; Dieb and Hafidi, 2013). These findings demonstrate that increased astrocyte-derived D-serine is involved in the modulation of orofacial posttraumatic neuropathic pain *via* acting on the NMDARs in the SpVc (Dieb and Hafidi, 2013).

Besides the SpVc, the NMDARs in the SpVi/c are also involved in the central sensitization related to chronic orofacial pain. Previous studies also have demonstrated that astroglial activation within the SpVi/c occurred following masseter or tooth pulp inflammation, which modulates the NMDAR phosphorylation *via* IL-1β derived from astroglial cells (Guo et al., 2007). It is reported that injection of the NMDAR antagonist into the SpVi/c attenuates masseter inflammatory hyperalgesia (Wang et al., 2006). Shimizu et al. (2009a) have reported that microinjection of IL-1β into the SpVi/c produces NMDAR-dependent orofacial hyperalgesia in rats. Guo et al. (2007) have reported that incubation of IL-1β in the medullary slices induced a significant and dose-dependent increase in P-NR1 levels in theSpVi/c. Microinjection of IL-1β into the SpVi/c *in vivo* also produces an increase in P-ser896-NR1 levels, which can be blocked by chelerythrine, a PKC inhibitor (Guo et al., 2007). Moreover, 2APB, a membrane permeable IP3 receptor antagonist also blocks IL-1β-induced NR1 phosphorylation. In addition, the PLC inhibitor U73122 and PLA2 inhibitor AACOCF3 can block the effect of IL-1β on NMDAR phosphorylation in SpVi/c (Guo et al., 2007). IL-1R colocalizes with the NR1 subunit in SpVi/c neurons, and the IL-1β signaling facilitates NMDAR activity in neurons (Guo et al., 2007). These findings demonstrate that the intracellular signal pathway of PLC, PLA2, and subsequent PKC activation is involved in the IL-1β-induced NR1 phosphorylation in the SpVi/c following peripheral nerve injury and inflammation, contributing to orofacial hyperalgesia.

### The central sensitization involved NMDARs in the brain

NMDAR-mediated pain is also found in advanced relays such as the ACC, forebrain, and medial prefrontal cortex (Wei et al., 2001; Medeiros et al., 2019). Wu et al. (2005) have demonstrated that the upregulation of NMDAR subunit NR2B in the ACC is related to inflammation-related persistent pain, and the local injection of antagonist in the ACC can significantly relieve pain hypersensitivity. Wei et al. (2001) found that mice with overexpression of NR2B in the forebrain showed stronger inflammation-related pain, the genetic modification of NMDAR in the forebrain can affect pain perception. In the experimental model of chronic neuropathic pain, Medeiros et al. (2019) have shown that blocking NMDAR in the prelimbic region of the medial prefrontal cortex can reduce chronic neuropathic pain while activating NMDAR can enhance neuropathic pain.

The cerebellar cortex consists of a molecular layer, Purkinje cell (PC) layer, and granule cell layer, which are mainly involved in molecular layer interneurons (MLIs), PC, granule cell (GC), and Golgi cell (Palay and Chan-Palay, 1974; Llinás, 2011). The various information coming from outside is transferred into the cerebellar cortex through the mossy fibers (MF)-GC-parallel fiber (PF) pathway and the climbing fibers (CF) pathway, which is involved in the control of sensory perception (Eccles et al., 1971; Palay and Chan-Palay, 1974; Bower and Woolston, 1983; Llinás, 2011). NMDARs are found on the membrane of GCs and MLIs, the terminal of PFs, and the postsynaptic membrane of CF-PC synapses (Glitsch and Marty, 1999; Casado et al., 2000). Electrophysiological recording *in vivo* demonstrates that pharmacologically blocking NMDARs abolish the facial stimulation evoked by GABAergic inhibitory components in the cerebellar cortical molecular layer (Xu et al., 2017). NR2A containing NMDARs contribute to the facial stimulation-evoked MF-GC synaptic transmission, suggesting that the NMDARs play an important role during the lateral sensory information synaptic transmission in the cerebellar granular layer *in vivo* in mice (Zhang et al., 2020). These findings suggest NMDARs in the cerebellar cortex may contribute to the initiation and development of chronic orofacial pain through mediating the synaptic transmission in the MF-GC-PF pathway and CF pathway, which are needed more preclinical studies to prove.

### The role of NMDARs in the descending nervous system

Activation of descending pathways is also involved in hyperalgesia in animal models of inflammatory and neuropathic pain (Porreca et al., 2002; Vanegas and Schaible, 2004; Kim Y. S. et al., 2014). Ferrari et al. (2014) have found that pretreatment of PAG with NMDAR antagonists can block the analgesic effect of electrical stimulation of the primary motor cortex, suggesting that the descending inhibition pathway depends on the function of NMDAR in the PAG (Porreca et al., 2002; Wang et al., 2019). Excitotoxic lesions of the RVM lead to significant attenuation of behavioral hyperalgesia after masseter inflammation, suggesting that modulatory inputs from the RVM enhance the hyperalgesia/allodynia after masseter inflammation (Sugiyo et al., 2005). Fields et al. (1995) have found that there are three types of neurons in RVM, namely, ON cell, OFF cell, and neutral cells, in which ON cells promote nociceptive sensation, while OFF cells inhibit nociceptive sensation. Therefore, RVM can produce two-way regulation by activating ON cells and OFF cells respectively. All three types of RVM cells expressed excitatory amino acid receptors and responded to exogenous glutamate (Heinricher and Roychowdhury, 1997). Current studies have found that NMDAR can produce two opposite effects in RVM. Heinricher et al. (2001) have found that the injection of NMDAR antagonist AP-5 into RVM attenuates the activation of OFF cells and the antinociceptive effect of morphine, so systemic administration of morphine could activate off cells in RVM through an NMDA-mediated excitation process, resulting in analgesic effect. Subsequent studies by Xu et al. (2007) have confirmed that secondary hyperalgesia induced by mustard oil is caused by continuous activation of ON cells mediated by NMDA.

## NMDARs Are Involved in The Chronic Orofacial Pain Associated with Psychological Stress and Depression

As psychological stress is closely related to the initiation and development of chronic orofacial pain (Huang et al., 2011; Okamoto et al., 2012), several studies focus on the role and mechanism of NDMARs in the masseter hyperalgesia induced by stress (Lin et al., 2019; Li et al., 2020). It is reported that activated astrocytes in the SpVc evoked by chronic restraint stress can release excessive IL-1β, followed by IL-1β binding to its specific receptor and promoting NMDAR phosphorylation in the neurons, ultimately contributing to masseter hyperalgesia (Zhao et al., 2015). Besides, activation of NR2B containing NMDARs also plays a key role in the masseter hyperalgesia induced by psychological stress though promoting phosphorylation of astrocytic JNK in the SpVc (Lin et al., 2019). These findings show that NMDARs are involved in the crosstalk between neurons and astrocytes, contributing to the pain hypersensitivity in the masseter induced by stress. Moreover, NMDAR-dependent central sensitization in the spinal dorsal horn and 5-HT-dependent descending facilitation also contribute to stress-induced spreading hyperalgesia in rats with orofacial inflammation (Li et al., 2020). Notably, increased NR1 receptors in the spinal dorsal horn mediate 5-HT-dependent descending pain facilitation from the RVM by regulating the expression of spinal 5-HT3 receptors, which contribute to behavioral hypersensitivity *via* a reciprocal neuron-glial signaling cascade (Guo et al., 2014). Therefore, increased NMDARs in the SpVc may also regulate 5-HT-dependent descending facilitation from RVM through regulating 5-HT3 receptors and contribute to chronic orofacial pain *via* cross-talk between neuron-glial cells. In addition, there is a reciprocal relationship between depressive behavior and TMJ inflammation-induced mechanical hyperalgesia. The expression of the MT1 receptor (melatonin receptor) is downregulated, whereas the expression of the NR1 subunit is upregulated in the SpVc of WKY rats with genetically predisposed depressive behavior. Intracisternal administration of 6-chloromelatonin (a melatonin analog) improves depressive behavior as well as mechanical hyperalgesia in WKY rats by preventing the upregulation of NR1 expression (Wang et al., 2012b). In summary, NMDARs play a vital role in the chronic orofacial pain associated with psychological stress and depression.

## The Sex-Related Difference in The Role and Mechanisms of NMDARs in Males and Females Under Chronic Orofacial Pain

It is widely known that there are sex differences in pain perception. For example, women with TMD reported lower experimental pain threshold and heat-pain tolerance limits along with greater severity of orofacial pain than their male counterparts (Ohrbach et al., 2011). However, it is still not fully clear, at present, what mechanisms may account for the sex-related difference in chronic orofacial pain. Some studies also have reported the effects of sex on the role and mechanism of NMDARs in chronic orofacial pain. There is a sex-related difference in the sensitivity of cutaneous mechanoreceptors to glutamate, a 4.5-fold lower EC50 for glutamate-induced mechanical sensitization in females as compared to male rats (Gazerani et al., 2010). Glutamate-induced cutaneous afferent fiber mechanical sensitization is significantly attenuated by co-administration of the NMDAR antagonist APV, which indicates that this effect is mediated, in part, through the activation of peripheral NMDARs (Gazerani et al., 2010). The NMDA-evoked afferent discharge is significantly greater in female than in male rats, which is resulted from an estrogen-mediated increase in the expression of peripheral NMDARs by masseter ganglion neurons in female rats (Dong et al., 2007). In addition, it is reported that estrogen can increase NMDAR-mediated neuronal responses through an effect on the NR2B subunit (Woolley et al., 1997; Foy et al., 1999). The sex-related difference in NMDAR expression and neuronal response in the masseter afferent fibers contribute to the greater sensitivity of NGF-induced mechanical sensitization to peripheral NMDAR antagonism in females than in male rats (Dong et al., 2007; Wong et al., 2014). However, no significant sex-related difference in the number of facial skin afferent fibers that expressed the NR2B subunit of the NMDAR was noted in the present study. The sex-related difference is also found in the mechanical sensitization induced by intramuscular injections of NGF into the masseter muscle in women and men. The mechanical sensitization is more pronounced in women than in men following injection of NGF into the masseter muscle (Svensson et al., 2003, 2008). Therefore, when we study the role and mechanisms of NMDARs related to chronic orofacial pain, sex differences should be taken into account. Furthermore, more studies are needed to investigate the mechanisms underlying the sex differences in the role of NMDARs in chronic orofacial pain.

## Effects of NMDAR Antagonists in The Management of Pain

As activation of NMDARs plays an important role in the initiation and development of chronic pain, especially neuropathic pain, NMDAR antagonists have garnered increasing attention over recent years as an adjunctive/alternative pain treatment modality (Collins et al., 2010; Kamp et al., 2019; Kreutzwiser and Tawfic, 2019). According to its action mode, NMDAR antagonists mainly include competitive antagonists, channel blockers, allosteric regulators, and uncoupling agents (Traynelis et al., 2010; Song et al., 2018; Hansen et al., 2021). Competitive antagonists inhibit the response of NMDARs to all agonists by competing with agonists or ligands that activate receptors. Typical competitive antagonists include (R)-AP5, CGP-78608, 7-CKA, and 5-dichlorouric acid (DCKA), etc. (Davies et al., 1981, 1982; Cai, 2006). However, due to the high conservation of GluN2 binding sites, competitive antagonists lack subunit selectivity (Furukawa et al., 2005; Kinarsky et al., 2005). Channel blockers are macromolecules that can combine with the channels of ion channels to block ion flow, such as ketamine, memantine, phencyclidine, and MK-801 (Song et al., 2018). Because the channel pores of NMDAR are relatively conservative, channel blockers only show moderate subunit selectivity for GluN2 subtypes (Dravid et al., 2007). However, partial capture blockers (trapping blockers) such as MK-801 and ketamine can preferentially inhibit the high activity of NMDAR, which will be beneficial to treatment, because the physiological level of NMDAR activity will not be affected (Chen and Lipton, 2005, 2006). Compared with antagonists and channel blockers, allosteric regulators have many advantages. First of all, allosteric regulators have two-way regulation, including positive and negative allosteric regulators (Yang and Svensson, 2008). Some allosteric regulators can bind to conservative low NMDAR sites, which is beneficial to the development of drugs with subunit selectivity (Luessen and Conn, 2022). Typical compounds include GluN2A selective negative allosteric regulator STCN-201, GluN1/2B receptor regulator isopropylphenidil, GluN2C/D selective regulator QNZ-46, and so on (Zhu et al., 2020). In addition, allosteric regulators do not affect the binding of orthomorphic ligands, allowing them to have synergistic or antagonistic effects with ligands, resulting in a variety of effects (Zhu and Paoletti, 2015). Unlike the previously mentioned strategy of directly acting on NMDARs, uncouplers regulate the signal cascade that mediates pain by interfering with proteins interacting downstream of NMDARs (Courtney et al., 2014; Sun et al., 2021). At present, many studies have confirmed that the NMDAR-PSD95-nNOS complex downstream of NMDARs is one of the important pathways that mediate pain sensitization (Chen et al., 2022; Huang et al., 2022). The formation of the PSD95-nNOS complex can be blocked by interfering peptides such as TAT-NR2B9c and TAT-GESV (Lai et al., 2014; Lee et al., 2018). Since NMDAR activity is not directly blocked, interfering peptides can inhibit hypersensitivity in pathological pain without changing the normal nociceptive sensation and avoid the side effects related to NMDAR antagonism (Aarts et al., 2002). However, compared with conventional chemical drugs, interfering peptides still have some problems, such as limited administration route, high manufacturing cost, and immunogenicity (Lai and Wang, 2010). At present, it has been found that small molecular compounds ZL006 and IC87201 can effectively destroy the effect of PSD95-nNOS interaction to simulate interfering peptides (Tochio et al., 2000; Lee et al., 2015). In addition, ZL006 can easily cross the blood-brain barrier during intravenous administration, making it a promising drug (Zhou et al., 2010; Chen et al., 2013).

Traditionally, NMDAR antagonists, such as ketamine, memantine, dextromethorphan, Mg^2+^, etc. have been used in the management of pain. Kreutzwiser and Tawfic (2019) have reviewed the pharmacotherapeutic profiles of four NMDAR antagonists, including ketamine, memantine, dextromethorphan, and Mg^2+^, and concluded that ketamine is the most studied NMDAR antagonist used to treat pain and possesses the most clinical utility (Kamp et al., 2019). Despite animal data suggesting some NMDAR antagonists are promising drugs for alleviating the development of chronic pain, we focused on their applications in humans and here we provide an overview of the NMDAR antagonists and the evidence for their use in various pain management settings (Table 1). Kamp et al. (2019) have updated the pharmacokinetic and pharmacodynamic considerations for ketamine in the treatment of chronic neuropathic pain in recent literature, which also supports that ketamine is a viable alternative in refractory neuropathic pain [e.g., post-herpetic neuralgia, complex regional pain syndrome (CRPS), central neuropathic pain, traumatic peripheral nerve pain, trigeminal neuropathic pain, spinal cord injury, phantom limb pain, painful limb ischemia, sickle cell pain, temporomandibular pain, fibromyalgia, migraine, and whiplash pain; Kamp et al., 2019; Kreutzwiser and Tawfic, 2019; Shiiba et al., 2021]. Moreover, Collins et al. (2010) have reviewed NMDAR antagonists (including ketamine, memantine, amantadine, dextromethorphan, MgSO_4_, MgCl_2_, Riluzole, GV 196771, and CNS5161 HCl ) for the treatment of neuropathic pain in clinical trials by meta-analysis. Their results demonstrate that no conclusions can yet be made about the efficacy of NMDAR antagonists on neuropathic pain, as positive and negative outcomes on pain relief are found. In addition, adverse events (including sedation, headache, dizziness, etc.) are also reported in the clinical trials for suppressing the physiological functions of NMDARs (Collins et al., 2010; Alviar et al., 2016). As the clinical trials reported in the available literature about the efficiency of NMDAR antagonists in chronic orofacial pain management is still limited, further large high-quality RCTs in clearly defined pain populations with chronic orofacial pain are required to support the firm conclusions on the role and safety of NMDAR antagonists in the management of chronic orofacial pain before widespread use.

**Table 1:** Summary of NMDAR antagonists for the treatment of pain in clinical trials.

Agent	Mechanism of action and receptor activity	Route	Condition
Ketamine	Ketamine binds non-competitively to an intra-channel site of the NMDAR to decrease channel opening time with high affinity (Schmid et al., 1999). The binding of ketamine at a second site located in the hydrophobic domain of the NMDAR decreases the frequency of channel opening (Schmid et al., 1999).	Intravenous (IV; Leung et al., 2001; Kvarnström et al., 2003, 2004; Gottrup et al., 2006; Niesters et al., 2013) Oral (Rigo et al., 2017) Topical (Gao et al., 2016; Peltoniemi et al., 2016; Dinis-Oliveira, 2017).	Chronic neuropathic pain (Niesters et al., 2013; Rigo et al., 2017) Post-nerve injury neuropathic pain (Leung et al., 2001; Gottrup et al., 2006) Postsurgical/posttraumatic neuropathic pain (Kvarnström et al., 2003) Spinal cord injury (Kvarnström et al., 2004)
Methadone	Antagonist to the NMDARs. Also binding primarily to the μ-opioid receptor (Aiyer et al., 2018).	Oral (Morley et al., 2003; Rigo et al., 2017)	Chronic neuropathic pain (Morley et al., 2003; Rigo et al., 2017)
Memantine	Memantine is an uncompetitive, low-moderate affinity NMDAR antagonist with strong voltage dependency and rapid unblocking kinetics (Witt et al., 2004)	Oral (Nikolajsen et al., 2000)	Post-nerve injury chronic pain (Nikolajsen et al., 2000)
Amantadine	Amantadine inhibits NMDAR un-competitively (Ossola et al., 2011) and stabilizes channel closure (Aiyer et al., 2018)	Oral (Aiyer et al., 2018) IV (Medrik-Goldberg et al., 1999)	Neuropathic pain (Medrik-Goldberg et al., 1999)
Dextromethorphan	Dextromethorphan is an uncompetitive, low-affinity NMDAR antagonist (Church et al., 1985; Aiyer et al., 2018)	Oral (McQuay et al., 1994; Heiskanen et al., 2002)	Neuropathic pain (McQuay et al., 1994; Carlsson et al., 2004) Chronic pain (Heiskanen et al., 2002) Facial neuralgias (Gilron et al., 2000)
Mg^2+^	Mg^2+^ is a physiological antagonist of the NMDAR ion channel (Srebro et al., 2017).	Oral (Pickering et al., 2011; Yousef and Al-deeb, 2013) IV (Felsby et al., 1996; Yousef and Al-deeb, 2013)	Neuropathic pain (Felsby et al., 1996; Pickering et al., 2011) Chronic low back pain (Yousef and Al-deeb, 2013)

In the aspect of new potential alternatives to NMDAR antagonists, a recent study has found botulinum toxin promotes orofacial antinociception by modulating TRPV1 and NMDARs in adult zebrafish (Rocha Barreto et al., 2022). Besides, the dietary constituent, resveratrol also has been reported to alleviate pain hypersensitivity by suppressing glutamatergic neurotransmission of the SpVcwide dynamic range neuron hyperexcitability *via* NMDARs under inflammatory and neuropathic conditions (Shimizu et al., 2009b; Wang et al., 2009; Takehana et al., 2017). These findings support botulinum toxin and resveratrol may be used as alternative medicines for the treatment of chronic orofacial pain. Notably, intravenous injection of bone marrow stromal cells (BMSCs) has shown potential to treat chronic pain including chronic orofacial pain (Huh et al., 2017; Guo et al., 2018). Application of BMSCs can attenuate pain hypersensitivity in the CCI-ION model (Guo et al., 2011), and reduce the amplitude and frequency of spontaneous excitatory postsynaptic currents in the SpVc of CCI-ION mice (Guo et al., 2016), suggesting BMSCs inhibit trigeminal neuronal hyperexcitability and primary afferent input by inhibiting the activation of NMDARs. In addition, NR2A tyrosine phosphorylation and PKCγ immunoreactivity in the RVM can be suppressed after BMSC application (Guo et al., 2016). Taken together, these findings indicate that BMSCs may be a new alternative used to treat chronic orofacial pain by reducing the NMDAR activities in the ascending and descending nervous system, which needs further studies to investigate the detailed mechanisms and evaluate the efficiency and safety.

Glutamate sensitizes sensory neurons to nociceptive stimulation in a manner dependent on NMDARs (Coderre and Melzack, 1992), The SGCs play an important role in this process by expressing glutamate transporters that regulate glutamate neurotransmission (Castillo et al., 2013), but SGCs also express Ca^2+^-permeable, Mg^2+^-insensitive NMDARs which are required for sensitization of neurons (Ferrari et al., 2014). Although the involvement of SGC NMDARs sensitization remains to be demonstrated with conditional silencing approaches *in vivo*, these findings highlight the possibility that glial NMDARs are important nociceptive intermediates. Indeed, it is extremely difficult to design drugs that target only glial cells without affecting neurons. Furthermore, the elimination of glial cells with glia-selective toxins may cause detrimental effects, given the supportive and protective roles of glia (Ji et al., 2013). While it is reported that administration of glial toxin to the DRG has been shown to reduce neuropathic pain (Liu et al., 2012). Besides, targeting SGC activation might prolong acute opioid analgesia (Berta et al., 2012). However, research about only targeting NMDAR in SGCs to treat chronic orofacial pain is rarely reported.

## Conclusions and Perspectives

In this review, we reviewed and elucidated the regulatory mechanisms of NMDAR and its role in the initiation, development, and treatment of chronic orofacial pain. NMDARs widely exist in the peripheral and central nervous system and participate in the ascending or descending regulation of chronic orofacial pain through a variety of molecules such as ions, amino acids, enzymes, and proteins, which provides many potential therapeutic targets for the strategy of chronic orofacial pain management through NMDARs. Therapeutic interventions on NMDARs have succeeded in alleviating orofacial inflammatory or neuropathic pain conditions, but we still need to search for more effective interventions. Because, at present, the effect of NMDAR antagonists is still controversial, and there are a series of side effects, its clinical application is limited. Meanwhile, different NMDAR subunits have different physiological functions in chronic orofacial pain. Studying the role of various NMDAR subunits in chronic orofacial pain may be beneficial to understand the pathogenesis of chronicorofacial pain. Therefore, further research on the regulatory mechanism of NMDARs after nerve injury or inflammation is helpful to develop new drugs to accurately regulate the pathological activities of NMDARs while ensuring their normal physiological function and reducing the side effects of drugs.

Moreover, the current studies about the role of NMDARs in the nociceptive circuits in the higher nervous centers are mostly based on spinal cord injury models, there are few studies on the role of NMDARs in the projection circuits based on orofacial pain models. Thus, more studies are needed to investigate the role and mechanism of NMDARs in the higher central nociceptive circuits in both ascending and descending nervous systems which regulate the nociceptive information from peripheral tissues of the trigeminal nervous system. In addition to the objective changes of tissue injury, inflammation, expression of acting factors, and pathway activation, the role of NMDARs in pain perception is also affected by psychological stress and depression, which suggests that there may be non-drug treatment strategies for chronic orofacial pain. Evidence indicated that there are sex-difference in the role of NMDARs in chronic orofacial pain, however, the detailed mechanisms underlying the regulation ofNMDARs on chronic orofacial pain are still not clear, which should be an important scientific topic in the future.

The limitations of this topical review are that even though we intend to focus on the role and regulatory mechanisms of NMDARs in the trigeminal nervous system in the development and maintenance of chronic orofacial pain including orofacial neuropathic and chronic orofacial inflammatory pain, about which there are still not many studies in the literature. Therefore, some summaries and discussions were based on the evidence found in the studies of the regulatory roles and mechanisms of NMDARs in the spinal nervous system in chronic pain.

In summary, there are many ways to regulate NMDARs, but the regulatory mechanism of NMDARs in the onset and development of chronic orofacial pain is not fully understood. Therefore, targeting upstream and downstream signal pathways of NMDARs may be a new approach to inhibit orofacial allodynia, hyperalgesia, and ectopic pain. This type of treatment is yet to be discovered and explored.

## Author Contributions

Y-JL and Y-LL contributed to the literature search, writing—original draft. Z-HF and H-LL drew schematic diagrams. Y-YZ and JL revised the manuscript. FL and J-FS contributed to conceptualization, supervision, revision, and resources. All authors contributed to the article and approved the submitted version.

## Funding

The study was funded by the National Natural Science Foundation of China (Grant Number: 81870800, 82071149), the Department of Science and Technology of Sichuan Province (Grant Number: 2021JDRC0166, 2020YJ0224), and the China Postdoctoral Science Foundation (Grant Number: 2020M683329).

## Conflict of Interest

The authors declare that the research was conducted in the absence of any commercial or financial relationships that could be construed as a potential conflict of interest.

## Publisher’s Note

All claims expressed in this article are solely those of the authors and do not necessarily represent those of their affiliated organizations, or those of the publisher, the editors and the reviewers. Any product that may be evaluated in this article, or claim that may be made by its manufacturer, is not guaranteed or endorsed by the publisher.
